# A 24-year longitudinal study of *Klebsiella pneumoniae* isolated from patients with bacteraemia and urinary tract infections reveals the association between capsular serotypes, antibiotic resistance, and virulence gene distribution

**DOI:** 10.1017/S0950268823001486

**Published:** 2023-08-07

**Authors:** Cheng-Yen Kao, Yen-Zhen Zhang, Carl Jay Ballena Bregente, Pei-Yun Kuo, Pek Kee Chen, Jo-Yen Chao, Tran Thi Thuy Duong, Ming-Cheng Wang, Tran Thi Dieu Thuy, Jazon Harl Hidrosollo, Pei-Fang Tsai, Ying-Chi Li, Wei-Hung Lin

**Affiliations:** 1 Institute of Microbiology and Immunology, College of Life Sciences, National Yang Ming Chiao Tung University, Taipei, Taiwan; 2 Division of Nephrology, Department of Internal Medicine, National Cheng Kung University Hospital, College of Medicine, National Cheng Kung University, Tainan, Taiwan; 3 Department of Pathology, National Cheng Kung University Hospital, College of Medicine, National Cheng Kung University, Tainan, Taiwan; 4 Department of Internal Medicine, College of Medicine, National Cheng Kung University Hospital, National Cheng Kung University, Tainan, Taiwan

**Keywords:** antimicrobial susceptibility, bacteraemia, capsule serotype, *K. pneumoniae*, urinary tract infection, virulence factor

## Abstract

Longitudinal studies on the variations of phenotypic and genotypic characteristics of *K. pneumoniae* across two decades are rare. We aimed to determine the antimicrobial susceptibility and virulence factors for *K. pneumoniae* isolated from patients with bacteraemia or urinary tract infection (UTI) from 1999 to 2022. A total of 699 and 1,267 *K. pneumoniae* isolates were isolated from bacteraemia and UTI patients, respectively, and their susceptibility to twenty antibiotics was determined; PCR was used to identify capsular serotypes and virulence-associated genes. K64 and K1 serotypes were most frequently observed in UTI and bacteraemia, respectively, with an increasing frequency of K20, K47, and K64 observed in recent years. *entB* and *wabG* predominated across all isolates and serotypes; the least frequent virulence gene was *htrA.* Most isolates were susceptible to carbapenems, amikacin, tigecycline, and colistin, with the exception of K20, K47, and K64 where resistance was widespread. The highest average number of virulence genes was observed in K1, followed by K2, K20, and K5 isolates, which suggest their contribution to the high virulence of K1. In conclusion, we found that the distribution of antimicrobial susceptibility, virulence gene profiles, and capsular types of *K. pneumoniae* over two decades were associated with their clinical source.

## Introduction

*K. pneumoniae* commonly causes antimicrobial-resistant opportunistic infections in hospitalised patients, in particular those with urinary tract infections (UTIs), pneumonia, intraabdominal infections, and bacteraemia. Infection is often associated with an impairment of the host defences due to underlying diseases such as malignancy, diabetes mellitus, chronic obstructive pulmonary disease, and alcoholism [1]. The pathogenicity of individual strains is attributed to the combination of various virulence factors such as adhesins (*fimH* and *mrkD*), capsule and mucoviscosity-associated proteins (*rmpA*, *rmpA2*, *magA*, and *wcaG*), and iron acquisition-related proteins (*iroB*, *iucA*, *peg344*, *kfuBC*, *ybtA*, and *entB*) [2, 3]. Capsular serotype K1 is relatively common in Taiwan and South Africa, but rarely seen in other countries [4, 5], and appears to play a significant role in the geographic restriction of *K. pneumoniae* infections.

Several antimicrobials (quinolones, aminoglycosides, third- and fourth-generation cephalosporins, carbapenems, and piperacillin or tazobactam) are the most commonly used agents for the treatment of *K. pneumoniae* infections. However, the emergence of multidrug-resistant (MDR) and extensively drug-resistant (XDR) strains has been widely reported in the past decade. Extended longitudinal studies on variations of the phenotypic and genetic characteristics of *K. pneumoniae* are rare, and hence, we investigated the distribution of antimicrobial susceptibility patterns, virulence gene factors, and K-type distribution of a large collection of isolates from patients with bacteraemia and UTI from 1999 to 2022.

## Methods

### Sampling and isolation of K. pneumoniae

The Institutional Review Board of National Cheng Kung University Hospital approved the research protocol of this study (IRB approval accession number A-ER-112-213). Nonduplicated *K. pneumoniae* stored isolates were randomly selected from patients with bacteraemia (699) or UTI (1,267) from 1999 to 2022. Isolates were identified by standard culture methods and a VITEK system (bioMérieux, Marcy-l’Etoile, France) and stored at −80 °C in Lysogeny broth (LB) containing 20% glycerol (v/v).

### Antimicrobial susceptibility testing

Antimicrobial susceptibility to 20 agents was determined with standard disc diffusion assays. Agents tested were amikacin (30 μg), amoxicillin/clavulanic acid (20/10 μg), ampicillin (10 μg), ampicillin/sulbactam (10/10 μg), cefazolin (30 μg), cefmetazole (30 μg), cefoxitin (30 μg), ceftazidime (30 μg), ceftriaxone (30 μg), ciprofloxacin (5 μg), colistin (10 μg), ertapenem (10 μg), gentamicin (10 μg), imipenem (10 μg), meropenem (10 μg), levofloxacin (5 μg), piperacillin/tazobactam (100/10 μg), sulfamethoxazole/trimethoprim (23.75/1.25 μg), tetracycline (30 μg), and tigecycline (15 μg) (BD BBL Sensi-Disc; Becton, Dickinson and Company, MD, USA). *E. coli* ATCC 25922 was used as a quality control strain. Susceptibility was determined according to the recommendations of the Clinical and Laboratory Standards Institute guidelines [6], and isolates were classified as MDR (non-susceptible to at least one agent in three or more antimicrobial categories), XDR (non-susceptible to at least one agent in all but two or fewer categories), or pandrug-resistant (PDR, non-susceptible to all antimicrobial agents) [7].

### Capsular type (K-type) determination

Genomic DNA of isolates was extracted by boiling methods and tested by polymerase chain reaction (PCR) for the presence of capsular serotype-specific (*cps*) genes (K1, K2, K5, K20, K47, K54, K57, and K64) [8]. The primers are listed in Supplementary Table S1.

### Detection of virulence-associated genes

Sixteen virulence-associated genes were tested (*iroB*, *iucA*, *peg589*, *peg1631*, *rmpA*, *ybtS*, *irp1*, *irp2*, *entB*, *wabG*, *kfuBC*, *ybtA*, *htrA*, *wcaG*, *mrkD*, and *allS*). Primer pairs, target genes, and PCR conditions are listed in Supplementary Table S1.

### Statistical analysis

Categorical variables were compared by the chi-square tests, and statistical analyses were conducted using SPSS version 22.0 (IBM, Armonk, NY, USA). A *P*-value of <0.05 indicated statistical significance.

## Results

### Demographic and clinical data from patients with bacteraemia and UTI due to K. pneumoniae

A total of 1,966 *K. pneumoniae* isolates were collected from patients at National Cheng Kung University Hospital who had bacteraemia or UTI from 1999 to 2022. Of these, 699 were from blood and 1,267 were from urine samples (Supplementary Table S2). We randomly grouped isolates at 5-year intervals to determine the change in strain characteristics over time. The majority of isolates (1,129) were obtained from female patients compared with male patients (837) (Supplementary Table S2). Likewise, the majority age range was 61 to 80 years with 105 (50.7%), 252 (48.3%), 142 (46.7%), 243 (42.1%), and 147 (41.3%) isolates recovered in 1999, 2004, 2009, 2014, and 2019–2022, respectively (Supplementary Table S2).

### Association of capsule serotypes and virulence factors with sample source

Among urine isolates, the most frequent capsular type of the eight screened was the K64 serotype (8.5%), followed by K2 (4.1%), K1 (3.1%), K20 (2.9%), K47 (1%), K57 (1%), and K5 (0.6%) (Supplementary Table S3). Correspondingly, the distribution among blood isolates was types K1 (9.6%), K64 (6.6%), and K2 (6.2%); the least frequent was K47 (0.7%) (Supplementary Table S3). Serotype K54 was not identified in either blood or urine samples. A significant difference in the frequency of serotype K1 was evident between blood and urine samples (*P* < 0.001).

The prevalence study of 16 virulence-associated genes among bacteraemia and UTI isolates showed *entB* (*P* = 0.419) and *wabG* (*P* = 0.056) to be predominant in both isolate groups (Supplementary Table S4). Nine genes, *iucA* (*P* < 0.001), *iroB* (*P* < 0.001), *irp1* (*P* < 0.001), *kfuBC* (*P* = 0.001), *rmpA* (*P* < 0.001), *wcaG* (*P* < 0.001), *peg1631* (*P* < 0.001), *peg589* (*P* < 0.001), and *allS* (*P* < 0.001), were more frequent in the blood isolates than in those from urine samples. Notably, the gene *htrA* was found more frequently in urine isolates than in those from blood samples (*P* < 0.001). The least frequently observed gene was *htrA* in bacteraemia isolates (Supplementary Table S4). The average number of virulence factor genes in bacteraemia and UTI isolates was 6.86 and 5.64 genes (*P* < 0.001), respectively.

### Antimicrobial susceptibility of K. pneumoniae and sample source

The susceptibility of all isolates to 20 antimicrobial agents grouped into 12 categories was examined to determine associations between antimicrobial susceptibility and their sample source (Supplementary Table S5). Most isolates were susceptible to amikacin, imipenem, meropenem, ertapenem, tigecycline, and colistin (Supplementary Table S5). However, there was a significant difference in their activity in the two isolate groups, except for ampicillin (*P* = 0.195), imipenem (*P =* 0.949), meropenem (*P =* 0.365), and colistin (*P =* 0.765). In general, urine isolates proved to be more resistant to most of the tested antibiotics compared with those from blood (Supplementary Table S5). Among blood isolates, 308 (44.1%) were classified as MDR and 9 (1.3%) XDR (Supplementary Table S6), while for urine isolates, 801 (63.2%) were MDR and 39 (3.1%) XDR. The difference in the prevalence rate of non-MDR, MDR, and XDR *K. pneumoniae* for the urine and blood isolates was statistically significant (*P* < 0.001). No PDR isolate was identified in the study.

### Change in prevalence of capsule serotype of bacteraemia and UTI K. pneumoniae over the study period

In 1999, serotype K1 was predominant in blood isolates, but in later years (2009, 2019–2022), it was replaced by the K64 serotype (Table 1). The latter serotype predominated in urine isolates over the two decades, except in 2004. The frequency of serotype K2 remained stable over the entire study period, in contrast to serotypes K5, K20, K47, and K57 (Table 1). A statistical analysis of the frequency distribution of the serotypes from bacteraemia and UTI patients at various intervals showed a significant difference in the occurrence of K1 and K20 in both groups of isolates (Table 1). Additionally, a significant difference was noted for types K1, K20, K57, and K64 in blood isolates and K1, K20, and K47 in urine isolates at various intervals (Table 1).

**Table 1.** Prevalence of capsule serotypes of *Klebsiella pneumoniae* isolated in blood and urine from 1999 to 2022

	Year of collection, *n* (%)											
Capsule type^a^	1999 (*n* = 207)		2004 (*n* = 522)		2009 (*n* = 304)		2014 (*n* = 577)		2019–2022 (*n* = 356)		*P*-value^b^	
	Blood (*n* = 139)	Urine (*n* = 68)	Blood (*n* = 193)	Urine (*n* = 329)	Blood (*n* = 84)	Urine (*n* = 220)	Blood (*n* = 249)	Urine (*n* = 328)	Blood (*n* = 34)	Urine (*n* = 322)	Blood	Urine
K1	17 (12.2)	0 (0)	15 (7.8)	18 (5.5)	1 (1.2)	1 (0.5)	34 (13.7)	16 (4.9)	0 (0)	4 (1.2)	0.002	<0.001
K2	7 (5.0)	3 (4.4)	13 (6.7)	16 (4.9)	4 (4.8)	5 (2.3)	18 (7.2)	13 (4.0)	1 (2.9)	15 (4.6)	0.772	0.620
K5	0 (0)	0 (0)	3 (1.6)	3 (0.9)	0 (0)	0 (0)	8 (3.2)	3 (0.9)	0 (0)	2 (0.6)	0.077	0.616
K20	0 (0)	0 (0)	16 (8.3)	15 (4.6)	4 (4.8)	12 (5.5)	12 (4.8)	6 (1.8)	0 (0)	4 (1.2)	0.006	0.005
K47	0 (0)	0 (0)	0 (0)	0 (0)	0 (0)	1 (0.5)	4 (1.6)	2 (0.6)	1 (2.9)	10 (3.1)	0.086	0.001
K57	1 (0.7)	0 (0)	10 (5.2)	8 (2.4)	0 (0)	2 (0.9)	1 (0.4)	1 (0.3)	0 (0)	2 (0.6)	0.001	0.053
K64	12 (8.6)	5 (7.3)	3 (1.6)	16 (4.9)	11 (13.1)	25 (11.4)	15 (6.0)	35 (10.7)	5 (14.7)	26 (8.1)	0.001	0.057
Others^c^	102 (73.4)	60 (88.2)	133 (68.9)	253 (76.9)	64 (76.2)	174 (79.1)	157 (63.1)	252 (76.8)	27 (79.4)	259 (80.4)	0.058	0.230

a No K54 *K. pneumoniae* was detected in this study.

b *P*-value is the statistical result of the comparison of the prevalence of K type in 1999, 2004, 2009, 2014, and 2019–2022 in blood or urine *K. pneumoniae* isolates.

c Others refers to isolates that have non-K1, non-K2, non-K5, non-K20, non-K47, non-K54, non-K57, or non-K64 capsule serotype.

### Prevalence of virulence-associated genes of K. pneumoniae in blood and urine isolates

The most predominant virulence-associated genes over the study period were *entB* and *wabG* (Table 2). In general, the prevalence of these genes was stable in blood and urine isolates. However, there was a marked decrease in genes *iucA*, *iroB*, *ybtS*, *kufBC*, *rmpA*, *wcaG*, *peg1631*, *peg589,* and *allS*, in blood isolates recovered in the final study period (2019–2022).

**Table 2.** Distribution of 17 virulence-associated genes in *Klebsiella pneumoniae* isolated from blood or urine from 1999 to 2022

	Year of collection											
Virulence genes	1999 (*n* = 207)		2004 (*n* = 522)		2009 (*n* = 304)		2014 (*n* = 577)		2019–2022 (*n* = 356)		*P*-value^a^	
	Blood (*n* = 139)	Urine (*n* = 68)	Blood (*n* = 193)	Urine (*n* = 329)	Blood (*n* = 84)	Urine (*n* = 220)	Blood (*n* = 249)	Urine (*n* = 328)	Blood (*n* = 34)	Urine (*n* = 322)	Blood	Urine
*Iron acquisition system*												
*iucA*	67 (48.2)	2 (2.9)	69 (35.8)	92 (28.0)	27 (32.1)	49 (22.3)	93 (37.3)	54 (16.5)	3 (8.8)	28 (8.6)	<0.001	<0.001
*iroB*	60 (43.2)	2 (2.9)	65 (33.7)	82 (24.9)	16 (19.1)	20 (9.1)	89 (35.7)	40 (12.2)	2 (5.9)	25 (7.7)	<0.001	<0.001
*entB*	119 (85.6)	54 (79.4)	192 (99.5)	319 (97.0)	84 (100)	220 (100)	249 (100)	327 (99.7)	33 (97.1)	315 (97.8)	<0.001	<0.001
*irp1*	53 (38.1)	10 (14.7)	83 (43.0)	101 (30.7)	28 (33.3)	76 (34.6)	112 (45.0)	89 (27.2)	12 (35.3)	100 (31.1)	0.297	0.024
*irp2*	53 (38.1)	28 (41.2)	66 (34.2)	104 (31.6)	33 (39.3)	77 (35.0)	116 (46.6)	150 (45.7)	19 (55.8)	158 (49.1)	0.032	<0.001
*ybts*	47 (33.8)	17 (25.0)	19 (9.8)	35 (10.6)	25 (29.8)	67 (30.5)	72 (28.9)	106 (32.3)	3 (8.8)	57 (17.7)	<0.001	<0.001
*kfuBC*	20 (14.4)	0 (0)	46 (23.8)	70 (21.3)	30 (35.7)	56 (25.5)	74 (29.7)	60 (18.3)	4 (11.8)	48 (14.9)	0.001	<0.001
*ybtA*	38 (27.3)	21 (30.9)	74 (38.3)	130 (39.5)	40 (47.6)	90 (40.9)	113 (45.4)	139 (42.4)	15 (44.1)	158 (49.1)	0.005	0.028
*Hypermucoviscosity*												
*rmpA*	62 (44.6)	1 (1.5)	63 (32.6)	79 (24.0)	17 (20.2)	24 (10.9)	90 (36.1)	49 (15.0)	2 (5.9)	22 (6.8)	<0.001	<0.001
*Capsule formation*												
*wabG*	127 (91.4)	63 (92.6)	186 (96.4)	322 (97.9)	82 (97.6)	219 (99.6)	245 (98.4)	314 (95.7)	34 (100)	322 (100)	0.005	<0.001
*wcaG*	23 (16.5)	55 (80.9)	86 (44.6)	94 (28.6)	12 (14.3)	30 (13.6)	68 (27.3)	82 (25.0)	3 (8.8)	57 (17.7)	<0.001	<0.001
*htrA*	1 (0.7)	0 (0)	14 (7.3)	29 (8.8)	8 (9.5)	20 (9.1)	10 (4.0)	110 (33.5)	1 (2.9)	45 (14)	0.016	<0.001
*Adhesins*												
*mrkD*	119 (85.6)	59 (86.8)	171 (88.6)	281 (85.4)	78 (92.9)	207 (94.1)	227 (91.2)	300 (91.5)	32 (94.1)	291 (90.4)	0.290	0.011
*Others*												
*peg1631*	39 (28.1)	19 (27.9)	32 (16.6)	42 (12.8)	23 (27.4)	39 (17.7)	114 (45.8)	49 (14.9)	3 (8.8)	21 (6.5)	<0.001	<0.001
*peg589*	53 (38.1)	4 (5.9)	46 (23.8)	39 (11.9)	17 (20.2)	30 (13.6)	74 (29.7)	43 (13.1)	3 (8.8)	25 (7.7)	0.001	0.076
*allS*	39 (28.1)	6 (8.8)	28 (14.5)	29 (8.8)	15 (17.9)	44 (20.0)	58 (23.3)	31 (9.5)	1 (2.9)	31 (9.6)	0.002	<0.001

a *P*-value is the statistical result of the comparison of the prevalence of virulence genes in 1999, 2004, 2009, 2014, and 2019–2022 in blood or urine *K. pneumoniae* isolates.

### Antibiotic susceptibility of bacteraemia and UTI K. pneumoniae isolates

Of the 20 antibiotic agents tested, ampicillin was consistently ineffective against all isolates over the study period (Table 3). In general, the activity of antibiotics decreased in UTI isolates compared with blood isolates throughout the study period, but unexpectedly, isolates from both sample groups showed higher resistance to most antibiotic agents in the period beginning in 2009 compared with the isolates from the other four time periods (Table 3). Moreover, the effectiveness of the carbapenems, imipenem, ertapenem, and meropenem, as well as tigecycline and colistin, decreased only in later years (Table 3).

**Table 3.** Distribution of antimicrobial non-susceptible *Klebsiella pneumoniae* isolated from blood or urine from 1999 to 2022

	Source (number of non-susceptible isolates, %)											
Antimicrobial category and agents	1999 (*n* = 207)		2004 (*n* = 522)		2009 (*n* = 304)		2014 (*n* = 577)		2019–2022 (*n* = 356)		*P*-value^a^	
	Blood (*n* = 139)	Urine (*n* = 68)	Blood (*n* = 193)	Urine (*n* = 329)	Blood (*n* = 84)	Urine (*n* = 220)	Blood (*n* = 249)	Urine (*n* = 328)	Blood (*n* = 34)	Urine (*n* = 322)	Blood	Urine
*Aminoglycoside*												
AN	6 (4.3)	19 (27.9)	9 (4.7)	23 (7.0)	15 (17.9)	54 (24.6)	11 (4.4)	26 (7.9)	1 (2.9)	12 (3.7)	<0.001	<0.001
GM	16 (11.5)	40 (58.8)	20 (10.4)	86 (26.1)	61 (72.6)	150 (68.2)	67 (26.9)	105 (32.0)	17 (50)	89 (27.6)	<0.001	<0.001
*Penicillins*												
AM	139 (100)	68 (100)	193 (100)	324 (98.5)	84 (100)	220 (100)	247 (99.2)	324 (98.8)	33 (97.1)	316 (98.1)	0.056	0.018
AMC	34 (24.5)	45 (66.2)	33 (17.1)	95 (28.9)	58 (69.1)	161 (73.2)	75 (30.1)	127 (38.7)	22 (4.7)	161 (50.0)	<0.001	<0.001
*Penicillins + β-lactamase inhibitors*												
SAM	18 (12.9)	48 (70.6)	43 (22.3)	122 (37.1)	65 (77.4)	183 (83.2)	96 (38.6)	145 (44.2)	24 (70.6)	160 (49.7)	<0.001	<0.001
TZP	1 (0.7)	15 (22.1)	12 (6.2)	35 (10.6)	42 (50.0)	94 (42.7)	53 (21.3)	103 (31.4)	14 (41.2)	80 (24.8)	<0.001	<0.001
*Carbapenems*												
IPM	0 (0)	0 (0)	1 (0.5)	1 (0.3)	0 (0)	0 (0)	13 (5.2)	15 (4.6)	5 (14.7)	19 (5.9)	<0.001	<0.001
ETP	1 (0.7)	2 (2.9)	3 (1.5)	5 (1.5)	2 (2.4)	14 (6.4)	17 (6.8)	43 (13.1)	2 (5.9)	42 (13.0)	0.008	<0.001
MEM	0 (0)	2 (2.9)	1 (0.5)	1 (0.3)	0 (0)	0 (0)	9 (3.6)	16 (4.9)	3 (8.8)	17 (5.3)	0.001	<0.001
*Non-extended spectrum cephalosporins*												
CZ	9 (6.5)	38 (55.9)	40 (20.7)	106 (32.2)	66 (78.6)	173 (78.6)	104 (41.8)	147 (44.8)	23 (67.6)	171 (53.1)	<0.001	<0.001
CMZ	2 (1.4)	15 (22.1)	16 (8.3)	39 (11.9)	25 (29.8)	76 (34.6)	44 (17.7)	87 (26.5)	12 (35.3)	117 (36.3)	<0.001	<0.001
*Extended spectrum cephalosporins*												
CRO	8 (5.8)	25 (36.8)	12 (6.2)	45 (13.7)	56 (66.7)	121 (55.0)	53 (21.3)	95 (29.0)	15 (44.1)	100 (31.1)	<0.001	<0.001
CAZ	6 (4.3)	19 (27.9)	21 (10.9)	67 (20.4)	56 (66.7)	124 (56.4)	71 (28.5)	113 (34.5)	19 (55.9)	138 (42.9)	<0.001	<0.001
*Cephamycins*												
FOX	6 (4.3)	27 (39.7)	19 (9.8)	58 (17.6)	38 (45.2)	104 (47.3)	54 (21.7)	113 (34.5)	14 (41.2)	118 (36.6)	<0.001	<0.001
*Fluoroquinolones*												
CIP	18 (12.9)	49 (72.1)	36 (18.7)	116 (35.3)	65 (77.4)	178 (80.9)	73 (29.3)	172 (52.4)	21 (61.8)	200 (62.1)	<0.001	<0.001
LVX	9 (6.5)	42 (61.8)	23 (11.9)	62 (18.8)	48 (57.1)	148 (67.3)	52 (20.9)	112 (34.1)	24 (70.6)	102 (31.7)	<0.001	<0.001
*Tetracyclines*												
TE	36 (25.9)	47 (69.1)	45 (23.3)	120 (36.5)	59 (70.2)	159 (72.3)	78 (31.3)	139 (42.4)	20 (58.8)	141 (43.8)	<0.001	<0.001
*Glycylcyclines*												
TIG	0 (0)	0 (0)	3 (1.6)	7 (2.1)	7 (8.3)	23 (10.5)	5 (2.0)	16 (4.9)	1 (2.9)	17 (5.3)	0.001	<0.001
*Folate pathway inhibitors*												
SXT	34 (24.5)	58 (85.3)	47 (24.4)	149 (45.3)	54 (64.3)	152 (69.1)	63 (25.3)	147 (44.8)	19 (55.9)	165 (51.2)	<0.001	<0.001
*Polymyxins*												
CL	0 (0)	0 (0)	2 (1.0)	5 (1.5)	0 (0)	4 (1.8)	1 (0.4)	3 (0.9)	3 (8.8)	3 (0.9)	<0.001	0.117

Abbreviations: AM, ampicillin; AMC, amoxicillin; AN, amikacin; CAZ, ceftazidime; CIP, ciprofloxacin; CL, colistin; CMZ, cefmetazole; CRO, ceftriaxone; CZ, cefazolin; ETP, ertapenem; FOX, cefoxitin; GM, gentamicin; IPM, imipenem; LVX, levofloxacin; MEM, meropenem; SAM, ampicillin or sulbactam; SXT, sulfamethoxazole or trimethoprim; TE, tetracycline; TIG, tigecycline; TZP, piperacillin or tazobactam.

a *P*-value is the statistical result of the comparison of the prevalence of antibiotic non-susceptible isolates in 1999, 2004, 2009, 2014, and 2019–2022 in blood or urine *K. pneumoniae* isolates.

### Capsular serotype is associated with virulence-associated genes and antimicrobial susceptibility

Capsular serotypes are associated with the phenotypes and virulence of *K. pneumoniae* [9]. Therefore, we explored the relationship between the virulence-associated genes (Table 4) and antimicrobial susceptibility (Table 5), with their serotypes. The genes *entB* and *wabG* were dominant for all capsular serotypes (Table 4). The distribution of the tested genes (except *entB*, *ybtA*, *wabG*, *htrA*, and *mrkD*) was significantly different among isolates of different capsular serotypes (Table 4). In addition, the highest average number of virulence genes was found in K1 isolates (11.42), followed by K2 (7.22) and K20 isolates (7.10) (Table 4).

**Table 4.** Prevalence of virulence factor genes in various capsular types of *Klebsiella pneumoniae*

	Capsular type^a^, *n* (%)									
Virulence genes	Others^b^ (*n* = 1481)	K1 (*n* = 106)	K2 (*n* = 95)	K5 (*n* = 19)	K20 (*n* = 69)	K47 (*n* = 18)	K57 (*n* = 25)	K64 (*n* = 153)	Total (1966)	*P*-value^c^
*Iron acquisition system*										
*iucA*	257 (17.6)	91 (73.4)	37 (38.9)	8 (42.1)	37 (53.6)	3 (16.7)	15 (60.0)	36 (23.5)	484 (24.6)	<0.001
*iroB*	207 (14.1)	88 (71.0)	34 (35.8)	7 (36.8)	21 (30.4)	2 (11.1)	15 (60.0)	27 (17.6)	401 (20.4)	<0.001
*entB*	1439 (97.2)	104 (98.1)	92 (96.8)	19 (100)	68 (98.6)	18 (100)	25 (100)	147 (96.1)	1912 (97.3)	0.848
*irp1*	411 (28.1)	91 (73.4)	42 (44.2)	7 (36.8)	49 (71.0)	8 (44.4)	4 (16.0)	52 (34.0)	664 (33.8)	<0.001
*irp2*	538 (36.8)	87 (70.2)	53 (55.8)	6 (31.6)	39 (56.5)	6 (33.3)	3 (12.0)	72 (47.1)	804 (40.9)	<0.001
*ybts*	321 (21.9)	27 (21.8)	34 (35.8)	2 (10.5)	12 (17.4)	5 (27.8)	5 (20.0)	42 (27.4)	448 (22.8)	0.038
*kfuBC*	271 (18.5)	73 (58.9)	18 (18.9)	5 (26.3)	5 (7.3)	1 (5.6)	1 (4.0)	34 (22.2)	408 (20.8)	<0.001
*ybtA*	604 (41.3)	57 (46.0)	46 (48.4)	11 (57.9)	25 (36.2)	6 (33.3)	10 (40.0)	59 (38.6)	818 (41.6)	0.812
*Hypermucoviscosity*										
*rmpA*	207 (14.1)	88 (71.0)	34 (35.8)	7 (36.8)	26 (37.7)	2 (11.1)	15 (60.0)	30 (19.6)	409 (20.8)	<0.001
*Capsule formation*										
*wabG*	1421 (97.1)	122 (98.4)	95 (100)	19 (100)	67 (97.1)	18 (100)	23 (92.0)	149 (97.4)	1914 (97.4)	0.336
*wcaG*	316 (21.6)	83 (66.9)	35 (36.8)	4 (21.1)	28 (40.6)	0 (0)	11 (44.0)	33 (21.6)	510 (25.9)	<0.001
*htrA*	185 (12.6)	10 (8.1)	13 (13.7)	3 (15.6)	5 (7.3)	2(11.1)	1(4.0)	19 (12.4)	238 (12.1)	0.555
*Adhesins*										
*mrkD*	1312 (89.7)	109 (87.9)	88 (92.6)	16 (84.2)	62 (89.9)	18 (100)	22 (88.0)	138 (90.2)	1765 (89.8)	0.764
*Others*										
*peg1631*	238 (16.3)	65 (52.4)	26 (27.4)	4 (21.1)	13 (18.8)	4 (22.2)	7 (28.0)	24 (15.7)	381 (19.4)	<0.001
*peg589*	168 (11.5)	86 (69.4)	23 (24.2)	4 (21.1)	23 (33.3)	1 (5.6)	2 (8.0)	27 (17.6)	334 (17.0)	<0.001
*allS*	188 (12.9)	48 (38.7)	12 (12.6)	5 (26.3)	5 (7.4)	1 (5.6)	4 (16.0)	19 (12.4)	282 (14.3)	<0.001
*Number of genes (SD)*	5.57 (2.08)	11.42 (2.37)	7.22 (2.58)	6.68 (1.73)	7.10 (2.35)	5.33 (2.57)	6.56 (2.00)	6.00 (2.42)	6.07 (2.55)	<0.001

a No K54 *K. pneumoniae* was detected in this study.

b Others refers to isolates that have non-K1, non-K2, non-K5, non-K20, non-K47, non-K54, non-K57, or non-K64 capsule serotype.

c *P*-value is the statistical result of the comparison of the prevalence of virulence genes in different capsular type *K. pneumoniae* isolates.

**Table 5.** Distribution of antimicrobial non-susceptible *Klebsiella pneumoniae* for various capsular types

	Capsular type^a^									
Antimicrobial category and agents	Others^b^ (*n* = 1481)	K1 (*n* = 106)	K2 (*n* = 95)	K5 (*n* = 19)	K20 (*n* = 69)	K47 (*n* = 18)	K57 (*n* = 25)	K64 (*n* = 153)	Total (1966)	*P*-value
*Aminoglycoside*										
AN	126 (8.5)	1 (1.0)	4 (4.2)	0 (0.0)	22 (31.9)	3 (16.7)	1 (4.0)	19 (12.4)	176 (9.0)	<0.001
GM	516 (34.8)	9 (8.5)	19 (20.0)	1 (5.3)	31 (44.9)	7 (38.9)	2 (8.0)	66 (43.1)	651 (33.1)	<0.001
*Penicillins*										
AM	1465 (98.9)	106 (100)	94 (99.0)	18 (94.7)	69 (100)	18 (100)	25 (100)	153 (100)	1948 (99.1)	0.525
AMC	636 (42.9)	12 (11.3)	35 (36.8)	3 (15.8)	33 (47.8)	7 (38.9)	5 (20.0)	80 (52.3)	811 (41.2)	<0.001
*Penicillins + β-lactamase inhibitors*										
SAM	720 (48.6)	12 (11.3)	31 (32.6)	3 (15.8)	34 (49.3)	8 (44.4)	6 (24.0)	90 (58.8)	904 (46.0)	<0.001
TZP	349 (23.6)	7 (6.6)	15 (15.8)	0 (0.0)	23 (33.3)	6 (33.3)	2 (8.0)	47 (30.7)	449 (22.8)	<0.001
*Carbapenems*										
IPM	41 (2.8)	1 (1.0)	3 (3.2)	0 (0.0)	3 (4.3)	2 (11.1)	0 (0.0)	4 (2.6)	54 (2.7)	0.490
ETP	94 (6.3)	2 (1.9)	7 (7.4)	0 (0.0)	4 (5.8)	4 (22.2)	2 (8.0)	18 (11.8)	131 (6.7)	0.014
MEM	36 (2.4)	1 (1.0)	3 (3.2)	0 (0.0)	2 (2.9)	3 (16.7)	1 (4.0)	3 (2.0)	49 (2.5)	0.010
*Non-extended spectrum cephalosporins*										
CZ	683 (46.1)	17 (16.0)	30 (31.6)	2 (10.5)	39 (56.5)	10 (55.6)	4 (16.0)	92 (60.1)	877 (44.6)	<0.001
CMZ	342 (23.1)	4 (3.8)	14 (14.7)	2 (10.5)	9 (13.0)	6 (33.3)	2 (8.0)	54 (35.3)	433 (22.0)	<0.001
*Extended spectrum cephalosporins*										
CRO	398 (26.9)	12 (11.3)	21 (22.1)	0 (0.0)	28 (40.6)	7 (38.9)	2 (8.0)	62 (40.5)	530 (27.0)	<0.001
CAZ	485 (32.7)	12 (11.3)	26 (27.4)	0 (0.0)	36 (52.2)	9 (50.0)	4 (16.0)	62 (40.5)	634 (32.2)	<0.001
*Cephamycins*										
FOX	439 (29.6)	6 (5.7)	18 (18.9)	2 (10.5)	17 (24.6)	7 (38.9)	2 (8.0)	60 (39.2)	551 (28.0)	<0.001
*Fluoroquinolones*										
CIP	727 (49.1)	15 (14.2)	34 (35.8)	3 (15.8)	39 (56.5)	12 (66.7)	6 (24.0)	92 (60.1)	928 (47.2)	<0.001
LVX	494 (33.4)	4 (3.8)	19 (20.0)	0 (0.0)	26 (37.7)	10 (55.6)	2 (8.0)	67 (43.8)	622 (31.6)	<0.001
*Tetracyclines*										
TE	670 (45.2)	12 (11.3)	30 (31.6)	2 (10.5)	33 (47.8)	7 (38.9)	3 (12.0)	87 (56.9)	844 (42.9)	<0.001
*Glycylcyclines*										
TIG	63 (4.3)	2 (1.9)	0	1 (5.3)	2 (2.9)	2 (11.1)	1 (4.0)	8 (5.2)	79 (4.0)	0.277
*Folate pathway inhibitors*										
SXT	704 (47.5)	8 (7.5)	35 (36.8)	2 (10.5)	35 (50.7)	8 (44.4)	5 (20.0)	91 (59.4)	888 (45.2)	<0.001
*Polymyxins*										
CL	13 (0.9)	1 (1.0)	1 (1.1)	1 (5.3)	1 (1.4)	0 (0.0)	1 (4.0)	3 (2.0)	21 (1.1)	0.231
*MDR/XDR* ^c^	884 (59.7)/ 36 (2.4)	19 (17.9)/ 1 (1.0)	45 (47.4)/ 1 (1.1)	3 (15.8)/ 0 (0)	40 (58.0)/ 1 (1.4)	5 (27.8)/ 4 (22.2)	5 (20.0)/ 1 (4.0)	108 (70.6)/ 4 (2.6)	1109 (56.4)/ 48 (2.4)	<0.001

Abbreviations: AM, ampicillin; AMC, amoxicillin; AN, amikacin; CAZ, ceftazidime; CIP, ciprofloxacin; CL, colistin; CMZ, cefmetazole; CRO, ceftriaxone; CZ, cefazolin; ETP, ertapenem; FOX, cefoxitin; GM, gentamicin; IPM, imipenem; LVX, levofloxacin; MEM, meropenem; SAM, ampicillin or sulbactam; SXT, sulfamethoxazole or trimethoprim; TE, tetracycline; TIG, tigecycline; TZP, piperacillin or tazobactam; MDR, multidrug-resistant; XDR, extensively drug-resistant.

a No K54 *K. pneumoniae* was detected in this study.

b Others refers to isolates that have non-K1, non-K2, non-K5, non-K20, non-K47, non-K54, non-K57, or non-K64 capsule serotype.

c Number of MDR isolates includes XDR isolates.

Among all capsule serotypes, K20, K47, and K64 showed widespread resistance to all antibiotic agents tested (Table 5). Moreover, of the seven serotypes listed, K20 showed the highest resistance rate to five agents (amikacin, 31.9%; gentamicin, 44.9%; piperacillin or tazobactam, 33.3%; ceftriaxone, 40.6%; and ceftazidime, 52.2%), while the rate for K47 varied between 11% (imipenem and tigecycline) and 66% (ciprofloxacin). Other associations of resistance rate with serotype K64 are listed in Table 5. Of the antimicrobials tested, only the susceptibility of ampicillin (*P =* 0.565), imipenem (*P =* 0.490), tigecycline (*P* = 0.277), and colistin (*P* = 0.234) did not show a significant difference between isolates of different capsular types. A significant difference in the prevalence of MDR and XDR *K. pneumoniae* in different capsule serotypes was noted (*P* < 0.001), but MDR strains were predominantly observed for unassigned serotypes (59.7%), K20 (58.0%), and K64 (70.6%), and the XDR phenotype for serotype K47 (22.2%) (Table 5).

## Discussion

In this study, we randomly selected 699 bacteraemia and 1,267 urinary isolates of *K. pneumoniae* and determined their capsular serotypes, virulence-associated genes, and susceptibility to 20 antibiotic agents. Previous studies have highlighted the association of serotypes K1 and K2 with these clinical sources [9, 10], which is consistent with our findings. However, these serotypes predominated only in the earlier years of the study period and were gradually replaced by strains of K64 after 2004 (Table 1). The latter serotype has been highly associated with carbapenem resistance [11, 12] and has been reported from several countries worldwide, as well as Taiwan [13]. In addition, the prevalence of this capsule serotype appears to be increasing. Zhou et al. [12] reported a subclonal shift in the dominant sequence type 11 carbapenem-resistant strains, whereby the previously prevalent K47 type had been replaced by the K64 serotype since 2016. Here, we found a marginal increase in the frequency of K64 in both bacteraemia and UTI isolates from 1999 to 2022 and also a similar increase in K47 isolates from 2009 to 2022. Further investigation of the epidemiological distribution of these serotypes is required.

Interestingly, unlike reported by others, we did not find the K54 serotype in isolates from either blood or urine [14, 15]. It is noteworthy that K54 strains have been linked predominantly with pulmonary diseases [10] and diabetes [15], which adds support to our findings with bacteraemic and UTI patients. Additionally, several other serotypes (K1, K2, K5, K20, K47, K54, K57, and K64) were notable by their absence or low frequency among 473 blood and 990 urine isolates. Although the urine isolates had fewer virulence genes (5.64 genes on average) compared with the blood isolates (6.86 genes on average), they were more resistant to antibiotics. These associations are worthy of further investigation.

The virulence factors, *entB* and *wabG*, in particular, were common in both groups of isolates. Although the high prevalence of *entB* could be used as an internal control in PCR assays [16], our data showed that 96.9% and 97.5% of blood and urine isolates had *entB.* Wu et al. [17] found that 96.65% of *K. pneumoniae* isolated from dairy cows in China had *entB*, and Mirzaie et al. [18] in Iran reported that only 80% of *K. pneumoniae* isolated from hospitalised patients harboured this gene. Therefore, the prevalence of *entB* in the species is variable among different sources and geographical origins of samples. The predominance of *wabG* – a gene encoding the biosynthesis of lipopolysaccharide in the outer cell membrane – has been reported in previous studies [19, 20]. Likewise, *entB* is involved in the synthesis of the iron acquisition siderophore of the species [21], and the presence of both of these virulence factors suggests that they play a critical role in colonisation and infection with *K. pneumoniae.* Likewise, the genes *rmpA*, *iroB*, and *iucA* are linked to the hypervirulent phenotype of *K. pneumoniae* [22], and moreover, these genes are located on virulence plasmids [23]. Our results showed a high prevalence rate of these three genes in K1 isolates compared with the other serotypes and thus may contribute to the hypervirulence of K1 isolates.

There was a high prevalence of MDR and XDR *K. pneumoniae* in our blood and urine isolates, which was consistent with previous studies [24, 25]. Furthermore, rates of ampicillin resistance throughout the study period were largely stable, whereas resistance to other antibiotics fluctuated over time, as recently recorded by Lin et al. [26]. Overall, resistance rates to the carbapenems, imipenem, meropenem, and colistin increased in later years.

Our findings of the correlation of virulence-associated genes and K types differ from the report of Hasani et al. [10] who reported *wcaG* and *rmpA* to be highly associated, respectively, with K20 and K54 isolates, whereas both genes were significantly associated with serotype K1 in our series. Nevertheless, our results are compatible with Rastegar et al. [27], who found the virulence genes *entB*, *mrkD*, *rmpA*, *iutA*, and *kfu* to be associated with K1 and K2 serotypes, particularly among the hypervirulent strain phenotype. ST23 is strongly associated with the K1 capsule type, and *allS* is a biomarker for K1-ST23, the most well-known hypervirulent lineage [28, 29]. However, the MLST types of *allS*-positive isolates in this study were not determined. We grouped all isolates of a single capsular type together to explore associations between capsular types and phenotypes, and virulence factor distribution. However, different genotypes within a capsular type can be markedly different in their characteristics, which may be dependent on the plasmids they carry. It follows that further genetic analysis of isolates by MLST or DNA macrorestriction typing is warranted to precisely characterise them. Finally, the distribution of capsular serotypes and their antibiotic susceptibility patterns showed an association of MDR and XDR strains with K20, K47, and K64 serotypes, which was consistent with the literature [13, 30, 31].

A possible limitation of our study is that all isolates originated from a single medical centre, which could inflate the number of isolates due to nosocomial transmissions. Likewise, the eight dominant serotypes were assigned by specific PCR assays, and therefore, the characteristics of isolates of other serotypes are unclear and worthy of future investigation.

In conclusion, we found that strain-defining characteristics of *K. pneumoniae* isolates differed between bacteraemia and UTI sources at various intervals over a 24-year period. Our results revealed interesting and sometimes novel associations between capsular serotypes, antibiotic resistance, and virulence gene distribution among the species.

## Supporting information

Kao et al. supplementary material Kao et al. supplementary material

## Data Availability

The other data will be made available on request.

## Supplementary material

For supplementary material accompanying this paper visit http://doi.org/10.1017/S0950268823001486. click here to view supplementary material
