# Supplementary material for: A 24-year longitudinal study of Klebsiella pneumoniae isolated from patients with bacteraemia and urinary tract infections reveals the association between capsular serotypes, antibiotic resistance, and virulence gene distribution
Source: Epidemiol Infect. 2023 Aug 7;151:e155. doi: 10.1017/S0950268823001486 (PMC10548544; doi:10.1017/S0950268823001486)
Supplement: Kao et al. supplementary material [file S0950268823001486sup001.doc]

**Table S1.** List of primers used in this study

| **Target** | **Primer sequence (5'-3')** | | **Function of gene** | **Annealing temperature (°C)** | **PCR product (bp)** | **References (for primer)** |
| --- | --- | --- | --- | --- | --- | --- |
| **K type detection** | | | | | | |
| K1 | | GTAGGTATTGCAAGCCATGC | capsular serotype K1 | 47 | 1047 | [1] |
|  |  | GCCCAGGTTAATGAATCCGT |  |  |  |  |
| K2 | | GGAGCCATTTGAATTCGGTG | capsular serotype K2 | 47 | 1121 | [1] |
|  |  | TCCCTAGCACTGGCTTAAGT |  |  |  |  |
| K5 | | GCCACCTCTAAGCATATAGC | capsular serotype K5 | 47 | 999 | [1] |
|  |  | CGCACCAGTAATTCCAACAG |  |  |  |  |
| K20 | | CCGATTCGGTCAACTAGCTT | capsular serotype K20 | 47 | 1116 | [1] |
|  |  | GCACCTCTATGAACTTTCAG |  |  |  |  |
| K47 | | GGACGCACAGTTTCCCAATTCGC | capsular serotype K47 | 47 | 389 | [2] |
|  |  | GCCCACATGAACCCACTTGGCA |  |  |  |  |
| K54 | | CATTAGCTCAGTGGTTGGCT | capsular serotype K54 | 47 | 881 | [1] |
|  |  | GCTTGACAAACACCATAGCAG |  |  |  |  |
| K57 | | CGACAAATCTCTCCTGACGA | capsular serotype K57 | 47 | 1182 | [1] |
|  |  | CGCGACAAACATAACACTCG |  |  |  |  |
| K64 | | TCAGTTCCGACCCTGATGCAGGTA | capsular serotype K64 | 47 | 247 | [2] |
|  |  | GCCAGAGCAACTATCATCCAAAGCCA |  |  |  |  |
| **Virulence factor detection** | | | | | | |
| *mrkD* | CCACCAACTATTCCCTCGAA | | adhesin [3, 4] | 60 | 226 | [5] |
|  | ATGGAACCCACATCGACATT | |  |  |  |  |
| *irp2* | GCTACAATGGGACAGCAACGAC | | yersiniabactin synthetases [6] | 59 | 230 | [7] |
|  | GCAGAGCGATACGGAAAATGC | |  |  |  |  |
| peg-589 | TGAACCCCTGAAGGTCTATC | | putative carboxymuconolactone decarboxylase family [8] | 55 | 236 | [9] |
|  | GTGATGAATAAACTACTGCGGC | |  |  |  |  |
| peg-1631 | GGGATTTATCAACCGCTTTG | | hypothetical protein [8] | 59 | 503 | [8] |
|  | TCTCCAGCATCATCGTCA | |  |  |  |  |
| *iroB* | CAAAAAAGCAGCAGAGGC | | salmochelin siderophore biosynthesis [8] | 59 | 671 | [8] |
|  | TCACTGGCGGAATCCAACAC | |  |  |  |  |
| *iucA* | GCT TAT TTC TCC CCA ACC C | | aerobactin siderophore biosynthesis [8] | 59 | 583 | [8] |
|  | TCA GCC CTT TAG CGA CAA G | |  |  |  |  |
|  | CAA TGA CGA GGG GGA TAA TC | |  |  |  |  |
| *rmpA* | GAG TAG TTA ATA AAT CAA TAG CAA T | | regulator of mucoid phenotype A [10, 11] | 60 | 205 | [8] |
|  | CAG TAG GCA TTG CAG CA | |  |  |  |  |
| *ybtS* | GACGGAAACAGCACGGTAAA | | yersiniabactin,  encoding salicylate synthase [12] | 60 | 242 | [13] |
|  | GAGCATAATAAGGCGAAAGA | |  |  |  |  |
| *magA* | GGTGCTCTTTACATCATTGC | | mucoviscosity-associated gene [11] | 60 | 1283 | [14] |
|  | GCAATGGCCATTTGCGTTAG | |  |  |  |  |
| *irp1* | TGA ATC GCG GGT GTC TTA TGC | | yersiniabactin biosynthesis [6] | 57 | 218 | [6] |
|  | TCC CTC AAT AAA GCC CAC GCT | |  |  |  |  |
| *entB* | CTG CTG GGA AAA GCG ATT GTC | | enterobactin biosynthesis [15] | 57 | 400 | [16] |
|  | AAG GCG ACT CAG GAG TGG CTT | |  |  |  |  |
|  | ACCATCGGCCATTTGATAGA | |  |  |  |  |
| *kfuBC* | GAAGTGACGCTGTTTCTGGC | | iron-uptake system [17] | 65 | 797 | [18] |
|  | TTTCGTGTGGCCAGTGACTC | |  |  |  |  |
| *ybtA* | TTACATCACGCGTTTAAAGG | | iron acquisition system [19] | 51 | 921 | [19] |
|  | ATGACGGAGTCACCGCAAAC | |  |  |  |  |
| *htrA* | CGTTCTGCCAGGATGGTTCT | | lipopolysaccharide and capsular polysaccharide [20] | 60 | 1071 | [21] |
|  | CCCCAATGATGACATCGCCT | |  |  |  |  |
| *wcaG* | GGTTGGKTCAGCAATCGTA | | capsule biosynthesis [4] | 53 | 169 | [19] |
|  | ACTATTCCGCCAACTTTTGC | |  |  |  |  |
| *allS* | CATTACGCACCTTTGTCAGC | | allantoin metabolism production [22] | 63 | 764 | [22] |
|  | GAATGTGTCGGCGATCAGCTT | |  |  |  |  |

**Table S2.** Distribution of sample source, gender, and age of patients, of *K. pneumoniae* collections

|  | **Year of collection, n (%)** | | | | |  |
| --- | --- | --- | --- | --- | --- | --- |
|  | 1999 (n=207) | 2004 (n=522) | 2009 (n=304) | 2014 (n=577) | 2019-2022 (n=356) | Total (n=1966) |
| **Sample source** | |  |  |  |  |  |
| Blood | 139 (67.1) | 193 (37.0) | 84 (27.6) | 249 (43.2) | 34 (9.6) | 699 (35.6) |
| Urine | 68 (32.9) | 329 (63.0) | 220 (72.4) | 328 (56.8) | 322 (90.4) | 1267 (64.4) |
| **Gender, n (%)** |  |  |  |  |  |  |
| Female | 97 (46.9) | 298 (57.1) | 182 (59.9) | 298 (51.6) | 254 (71.3) | 1129 (57.4) |
| Male | 110 (53.1) | 224 (42.9) | 122 (40.1) | 279 (48.4) | 102 (28.7) | 837 (42.6) |
| **Age, mean (SD)** | 58.4 (20.0) | 62.0 (20.4) | 68.4 (16.5) | 66.9 (19.7) | 67.9 (22.5) | 65.1 (20.2) |
| <=3 | 7 (3.4) | 13 (2.5) | 4 (1.3) | 12 (2.1) | 16 (4.5) | 52 (2.6) |
| 4-20 | 8 (3.9) | 17 (3.3) | 1 (0.3) | 12 (2.1) | 7 (2.0) | 45 (2.3) |
| 21-40 | 16 (7.7) | 42 (8.0) | 9 (2.9) | 33 (5.7) | 11 (3.1) | 111 (5.6) |
| 41-60 | 56 (27.1) | 123 (23.6) | 76 (25.0) | 111 (19.2) | 56 (15.7) | 422 (21.5) |
| 61-80 | 105 (50.7) | 252 (48.3) | 142 (46.7) | 243 (42.1) | 147 (41.3) | 889 (45.2) |
| >80 | 15 (7.2) | 75 (14.4) | 72 (23.7) | 166 (28.8) | 119 (33.4) | 447 (22.7) |

**Table S3. Distribution of capsular types of *K. pneumoniae* isolated from blood or urine in patients**

|  | **Source, n (%)** | |  |
| --- | --- | --- | --- |
| **Capsule type**^a^ | Blood (n=699) | Urine (n=1267) | *p*-value |
| K1 | 67 (9.6) | 39 (3.1) | <0.001 |
| K2 | 43 (6.2) | 52 (4.1) | 0.043 |
| K5 | 11 (1.6) | 8 (0.6) | 0.041 |
| K20 | 32 (4.6) | 37 (2.9) | 0.056 |
| K47 | 5 (0.7) | 13 (1.0) | 0.489 |
| K57 | 12 (1.7) | 13 (1.0) | 0.191 |
| K64 | 46 (6.6) | 108 (8.5) | 0.125 |
| Others^b^ | 483 (69.1) | 998 (78.8) | <0.001 |

^a^No K54 isolate was detected in this study.

^b^Others stands for isolates have non-K1, K2, K5, K20, K47, K54, K57, or K64 capsular serotype.

**Table S4.** **Distribution of 17 virulence-associated genes in *K. pneumoniae* isolated from blood or urine in patients**

|  | **Source, n (%)** | |  |
| --- | --- | --- | --- |
|  | Blood (n=699) | Urine (n=1267) | *p*-value |
| **Virulence genes** |  |  |  |
| **Iron-acquisition system** | |  |  |
| *iucA* | 259 (37.1) | 225 (20.1) | <0.001 |
| *iroB* | 232 (33.2) | 169 (13.3) | <0.001 |
| *entB* | 677 (96.9) | 1235 (97.5) | 0.419 |
| *irp1* | 288 (41.2) | 376 (29.7) | <0.001 |
| *irp2* | 287 (41.1) | 517 (40.8) | 0.913 |
| *ybts* | 166 (23.7) | 282 (22.3) | 0.451 |
| *kfuBC* | 174 (24.9) | 234 (18.4) | 0.001 |
| *ybtA* | 280 (40.1) | 538 (42.5) | 0.300 |
| **Hypermucoviscosity** |  |  |  |
| *rmpA* | 234 (33.5) | 175 (13.8) | <0.001 |
| **Capsule formation** |  |  |  |
| *wabG* | 674 (96.4) | 1,240 (97.9) | 0.056 |
| *wcaG* | 224 (32.0) | 286 (22.6) | <0.001 |
| *htrA* | 34 (4.9) | 204 (16.1) | <0.001 |
| **Adhesins** |  |  |  |
| *mrkD* | 627 (89.7) | 1138 (89.8) | 0.934 |
| **Others** |  |  |  |
| *peg1631* | 211 (30.2) | 170 (13.4) | <0.001 |
| *peg589* | 193 (27.6) | 141 (11.1) | <0.001 |
| *allS* | 141 (20.2) | 141 (11.1) | <0.001 |
| **Number of genes, (SD)** | 6.86 (2.94) | 5.64 (2.19) | <0.001 |

**Table S5. Distribution of antibiotic non-susceptible isolates in *K. pneumoniae* isolated from blood or urine in patients**

| **Antimicrobial category and agents** | **Source (number of non-susceptible isolates, %)** | |  |
| --- | --- | --- | --- |
|  | Blood (n=699) | Urine (n=1267) | *p*-value |
| **Aminoglycoside** |  |  |  |
| AN | 42 (6.0) | 134 (10.6) | 0.003 |
| GM | 181 (25.9) | 470 (37.1) | <0.001 |
| **Penicillins** |  |  |  |
| AM | 696 (99.6) | 1252 (98.8) | 0.195 |
| AMC | 222 (31.8) | 589 (46.5) | <0.001 |
| **Penicillins + β-lactamase inhibitors** | |  |  |
| SAM | 246 (35.2) | 658 (51.9) | <0.001 |
| TZP | 122 (17.5) | 327 (25.8) | <0.001 |
| **Carbapenems** |  |  |  |
| IPM | 19 (2.7) | 35 (2.8) | 0.949 |
| ETP | 25 (3.6) | 106 (8.4) | <0.001 |
| MEM | 13 (1.9) | 36 (2.8) | 0.365 |
| **Non-extended-spectrum cephalosporins** | |  |  |
| CZ | 242 (34.6) | 635 (50.1) | <0.001 |
| CMZ | 99 (14.2) | 334 (26.4) | <0.001 |
| **Extended-spectrum cephalosporins** | |  |  |
| CRO | 144 (20.6) | 386 (30.5) | <0.001 |
| CAZ | 173 (24.7) | 461 (36.4) | <0.001 |
| **Cephamycins** |  |  |  |
| FOX | 131 (18.7) | 420 (33.1) | <0.001 |
| **Fluoroquinolones** |  |  |  |
| CIP | 213 (30.5) | 715 (56.4) | <0.001 |
| LVX | 156 (22.3) | 466 (36.8) | <0.001 |
| **Tetracyclines** |  |  |  |
| TE | 238 (34.0) | 606 (47.8) | <0.001 |
| **Glycylcyclines** |  |  |  |
| TIG | 16 (2.3) | 63 (5.0) | 0.002 |
| **Folate pathway inhibitors** | |  |  |
| SXT | 217 (31.0) | 671 (53.0) | <0.001 |
| **Polymyxins** |  |  |  |
| CL | 6 (0.9) | 15 (1.2) | 0.765 |

AM, ampicillin; AMC, amoxicillin; AN, amikacin; CAZ ceftazidime; CIP, ciprofloxacin; CL, colistin; CMZ, cefmetazole; CRO, ceftriaxone; CZ, cefazolin; ETP, ertapenem; FOX, cefoxitin; GM, gentamicin; IPM, imipenem; LVX, levofloxacin; MEM, meropenem; SAM, ampicillin/sulbactam; SXT, sulfamethoxazole/trimethoprim; TE, tetracycline; TIG, tigecycline; TZP, piperacillin/tazobactam.

**Table S6. Distribution of MDR, XDR, and PDR-isolates in *K. pneumoniae* isolated from blood or urine in patients**

| **Antimicrobial category and agents** | **Source (number of non-susceptible isolates, %)** | |  |
| --- | --- | --- | --- |
|  | Blood (n=699) | Urine (n=1267) | *p*-value |
| Non-MDR | 382 (54.6) | 427 (33.7) | <0.001 |
| MDR^a^ | 308 (44.1) | 801 (63.2) |  |
| XDR | 9 (1.3) | 39 (3.1) |  |

^a^Number of MDR isolates includes XDR isolates. No PDR isolate was identified in this study.

MDR, multidrug-resistant; XDR, extensively drug-resistant; PDR, pandrug-resistant.

**References:**

(1) **Liu C, Guo J.** Hypervirulent Klebsiella pneumoniae (hypermucoviscous and aerobactin positive) infection over 6 years in the elderly in China: antimicrobial resistance patterns, molecular epidemiology and risk factor. *Ann Clin Microbiol Antimicrob* 2019; **18**(1): 4.

(2) **Wei T, et al.** Emergence of Hypervirulent ST11-K64 Klebsiella pneumoniae Poses a Serious Clinical Threat in Older Patients. *Front Public Health* 2022; **10**: 765624.

(3) **Sebghati TAS, et al.** Characterization of the type 3 fimbrial adhesins of Klebsiella strains. *Infection and immunity* 1998; **66**(6): 2887-2894.

(4) **Candan ED, Aksöz N.** Klebsiella pneumoniae: characteristics of carbapenem resistance and virulence factors. *Acta Biochimica Polonica* 2015; **62**(4).

(5) **Sahly H, et al.** Extended-spectrum β-lactamase production is associated with an increase in cell invasion and expression of fimbrial adhesins in Klebsiella pneumoniae. *Antimicrobial agents and chemotherapy* 2008; **52**(9): 3029-3034.

(6) **Pelludat C, Hogardt M, Heesemann Jr.** Transfer of the core region genes of the Yersinia enterocolitica WA-C serotype O: 8 high-pathogenicity island to Y. enterocolitica MRS40, a strain with low levels of pathogenicity, confers a yersiniabactin biosynthesis phenotype and enhanced mouse virulence. *Infection and immunity* 2002; **70**(4): 1832-1841.

(7) **Russo TA, et al.** Aerobactin mediates virulence and accounts for increased siderophore production under iron-limiting conditions by hypervirulent (hypermucoviscous) Klebsiella pneumoniae. *Infection and immunity* 2014; **82**(6): 2356-2367.

(8) **Russo TA, et al.** Identification of biomarkers for differentiation of hypervirulent Klebsiella pneumoniae from classical K. pneumoniae. *Journal of clinical microbiology* 2018; **56**(9): e00776-00718.

(9) **Yang Y, et al.** Clinical and microbiological characteristics of hypervirulent Klebsiella pneumoniae (hvKp) in a hospital from North China. *The Journal of Infection in Developing Countries* 2020; **14**(06): 606-613.

(10) **Turton JF, et al.** PCR characterization and typing of Klebsiella pneumoniae using capsular type-specific, variable number tandem repeat and virulence gene targets. *Journal of medical microbiology* 2010; **59**(5): 541-547.

(11) **Hartman LJ, et al.** Rapid real-time PCR assays for detection of Klebsiella pneumoniae with the rmpA or magA genes associated with the hypermucoviscosity phenotype: screening of nonhuman primates. *The Journal of Molecular Diagnostics* 2009; **11**(5): 464-471.

(12) **Lawlor MS, O'Connor C, Miller VL.** Yersiniabactin is a virulence factor for Klebsiella pneumoniae during pulmonary infection. *Infect Immun* 2007; **75**(3): 1463-1472.

(13) **Zhou M, et al.** Epidemiology and molecular characteristics of the type VI secretion system in Klebsiella pneumoniae isolated from bloodstream infections. *Journal of Clinical Laboratory Analysis* 2020; **34**(11): e23459.

(14) **Fang C-T, et al.** A novel virulence gene in Klebsiella pneumoniae strains causing primary liver abscess and septic metastatic complications. *The Journal of experimental medicine* 2004; **199**(5): 697-705.

(15) **Ferreira RL, et al.** High prevalence of multidrug-resistant Klebsiella pneumoniae harboring several virulence and β-lactamase encoding genes in a Brazilian intensive care unit. *Frontiers in microbiology* 2019; **9**: 3198.

(16) **Wasfi R, Elkhatib WF, Ashour HM.** Molecular typing and virulence analysis of multidrug resistant Klebsiella pneumoniae clinical isolates recovered from Egyptian hospitals. *Sci Rep* 2016; **6**: 38929.

(17) **Ma L-C, et al.** Genomic heterogeneity in Klebsiella pneumoniae strains is associated with primary pyogenic liver abscess and metastatic infection. *The Journal of infectious diseases* 2005; **192**(1): 117-128.

(18) **Brisse S, et al.** Virulent clones of Klebsiella pneumoniae: identification and evolutionary scenario based on genomic and phenotypic characterization. *PLoS One* 2009; **4**(3): e4982.

(19) **Zhang S, et al.** Phenotypic and genotypic characterization of Klebsiella pneumoniae isolated from retail foods in China. *Frontiers in microbiology* 2018; **9**: 289.

(20) **Cortés G, et al.** Role of the htrA gene in Klebsiella pneumoniae virulence. *Infect Immun* 2002; **70**(9): 4772-4776.

(21) **Mirzaie A, Ranjbar R.** Antibiotic resistance, virulence-associated genes analysis and molecular typing of Klebsiella pneumoniae strains recovered from clinical samples. *AMB Express* 2021; **11**(1): 122.

(22) **Lan Y, et al.** Prevalence of pks gene cluster and characteristics of Klebsiella pneumoniae‐induced bloodstream infections. *Journal of clinical laboratory analysis* 2019; **33**(4): e22838.
